# The Workwell trial: protocol for the process evaluation of a randomised controlled trial of job retention vocational rehabilitation for employed people with inflammatory arthritis

**DOI:** 10.1186/s13063-022-06871-z

**Published:** 2022-11-09

**Authors:** Alison Hammond, Kathryn A. Radford, Angela Ching, Yeliz Prior, Rachel O’Brien, Sarah Woodbridge, June Culley, Jennifer Parker, Paula Holland

**Affiliations:** 1 Centre for Human Movement and Rehabilitation, University of Salford, Allerton, Frederick Road, Salford, Greater Manchester M6 6PU UK; 2 Division of Rehabilitation and Ageing, University of Nottingham, Nottingham, UK; 3 College of Health, Well Being and Life Sciences, Sheffield Hallam University, Sheffield, UK; 4 Patient research partner, Derbyshire, UK; 5 Division of Health Research, Lancaster University, Lancaster, UK

**Keywords:** Process evaluation, Complex intervention, Mixed methods, Arthritis, Vocational rehabilitation, Occupational therapy, Work, Protocol, Randomised controlled trial

## Abstract

**Background:**

The Workwell trial is a multi-centre randomised controlled trial with the aims of evaluating the effectiveness and cost-effectiveness of job retention vocational rehabilitation for employed people with inflammatory arthritis, who are experiencing work difficulties due to their arthritis. Vocational rehabilitation is delivered by health service occupational therapists, who have received additional training in providing this Workwell intervention. A process evaluation will be undertaken alongside the main trial to investigate implementation fidelity; understand key stakeholders’ perspectives of the intervention and the social and structural context in which the intervention is provided; and explore issues related to future implementation in clinical practice. This protocol describes the aims, objectives, and methodology of the Workwell trial process evaluation.

**Methods:**

This mixed methods process evaluation will follow the Medical Research Council’s Guidance on process evaluations for complex interventions. It will be underpinned by the conceptual framework for implementation fidelity (CFIF) and normalisation process theory (NPT). We will analyse treatment records, work assessments, and treatment notes to ascertain implementation fidelity. Semi-structured interviews with trial participants, their employer/line managers, treating therapists, and their therapy service managers will be undertaken to explore perceptions of the intervention, contextual factors, and potential for future implementation in practice. Interview topic guides will be informed by NPT. Therapists’ views about Workwell training will be explored via questionnaires following training, and interviews and focus groups following treatment delivery to inform future implementation. Quantitative data will be analysed descriptively. Qualitative data will be analysed using thematic analysis. NPT will guide data analysis and interpretation. Findings from the different elements of this embedded design process evaluation will be reported separately and then the elements integrated. The process evaluation data will be analysed independently of the Workwell trial outcome evaluation. The process evaluation data will then be reviewed in the light of the trial findings.

**Discussion:**

Few trials of job retention vocational rehabilitation in arthritis have included process evaluations. This process evaluation will assist in understanding factors influencing trial outcomes and identifying potential contextual barriers and facilitators for the potential implementation of Workwell vocational rehabilitation into clinical services.

**Trial registration:**

ClinicalTrials.gov NCT03942783. Registered on 08 May 2019. ISRCTN Registry ISRCTN61762297. Registered on 13 May 2019. Retrospectively registered.

**Supplementary Information:**

The online version contains supplementary material available at 10.1186/s13063-022-06871-z.

## Background

Work problems are common amongst people with inflammatory arthritis (IA) (e.g. rheumatoid arthritis (RA), psoriatic arthritis (PsA)). These include work instability (i.e. a mismatch between abilities and job demands), presenteeism (i.e. reduced work productivity), and work disability (i.e. job loss due to arthritis). Amongst those with RA, 67% experience presenteeism, 10% stop working within 2 years, and 50% within 4.5 to 22 years of diagnosis [1–3]. Job retention vocational rehabilitation (JRVR) provides support to employed people who are working but experiencing difficulties with work because of their health problem. JRVR can potentially prevent or postpone work disability and reduce presenteeism through work assessment, work-related rehabilitation, and modifying work and/or the workplace to suit the person’s condition and abilities (i.e. job accommodations) [4].

A systematic review of JRVR trials, including people with IA, identified six trials of acceptable quality: two each conducted in the Netherlands, United States (US), and United Kingdom (UK), with varying results [4, 5]. Both Dutch trials, conducted from hospital rheumatology departments, resulted in no significant differences in work outcomes, compared to control groups receiving usual care. The first (*n* = 140) recruited people with concerns about job loss. It included a 4-to-12-week programme of comprehensive disease and work assessments; multidisciplinary rehabilitation; education about work disability benefits; and vocational counselling and advice/guidance about relevant job accommodations, with a final report of recommended job accommodations sent to the occupational health physician at the participant’s workplace. Dutch companies must legally have a contract with an occupational health physician, who then co-ordinates relevant job accommodations, funded by the employer [6]. Although there were significant improvements in fatigue and mental health, the authors suggested the lack of work outcome changes could be due to issues with implementing job accommodations, as co-operation with some companies’ occupational health services was “troublesome”; and recruiting some 40% of participants on long-term sick leave, potentially meaning the intervention was already too late for many [6]. The second trial (*n*=150) sought to overcome these issues by recruiting people with RA reporting difficulties in work functioning, excluding those on sick leave and involving key actors in the workplace [7]. JRVR was co-ordinated by a care manager (an occupational health physician) who identified work issues, liaised with the rheumatology team, including rheumatologists, a work rehabilitation-trained occupational therapist, and directly with the occupational health physician at the participant’s workplace. The therapist conducted a workplace assessment identifying work problems, discussed feasible solutions with the participant and their supervisor, and agreed an action plan, which the participant and supervisor were responsible for implementing. A summary report was provided to participant, supervisor, workplace occupational health physician, and the team. An additional work visit could be conducted if ergonomic training was required when job accommodations were in place. The care manager evaluated JRVR progress with the participant at 6 and 12 weeks. At 12 months, there were no effects on work, pain, fatigue, or quality of life. The authors suggested that too few participants had a moderate-high risk of work instability, as only half had RA-Work Instability Scale (RA-WIS) scores of ≥ 10, i.e. indicating need for JRVR [8]. Many participants and supervisors, therefore, may not have perceived the need to make work changes as yet [7].

The two trials in the US both had positive work outcomes, compared to control groups receiving usual care and written work information. Both included people with IA and osteoarthritis (OA) with “concerns about health affecting ability to work now or in the next few years,” recruited via rheumatology departments and the community [9, 10]. In the first trial (*n* = 242), the intervention was provided by experienced vocational rehabilitation (VR) counsellors, who conducted two 1.5-h meetings, spread over several months, including a detailed work assessment (the Work Experience  Survey- Rheumatic Conditions: WES-RC) [11]. From this, recommendations for job accommodations and relevant resources were identified, vocational counselling, education on legal rights for work accommodations, and skills training to request these were provided. No workplace visits were conducted. In the US, employees are responsible for requesting job accommodations with their employers, who are then responsible for funding accommodations, although there may be some State VR Agency financial support. At 4-year follow-up, there were fewer job losses, with longer time to job loss, in the intervention compared to the control group [9]. In the second trial (*n* = 287), the same intervention was delivered by additionally work rehabilitation-trained occupational therapists and physiotherapists. They also conducted the WES-RC, in one 1.5-h meeting, identifying solutions and job accommodations, with a written action plan developed for participant and therapist to follow. Therapists then telephoned participants at 3 weeks and 3 months post-assessment to identify progress and recommend further solutions, if needed. At 2 years, there were also fewer job losses, and longer time to job loss, in the intervention compared to the control group, although no differences in work ability [10].

The trials in the UK also had positive results but both were small. The first (a proof-of-concept trial, *n*=32) recruited people with RA and with RA-WIS scores ≥ 10. The intervention included a comprehensive work, function, and psychosocial assessment, with individualised treatment including job accommodation recommendations, education on legal rights and requesting job accommodations, a comprehensive occupational therapy programme including ergonomic, fatigue, and stress management training, daily living training, exercise, splinting and referral to other multidisciplinary team members (e.g. physiotherapy, podiatry if relevant), and a group self-management education meeting, as appropriate. A work site visit was offered if relevant (6/16 participants). In the UK, the employee normally requests job accommodations from their employer (via their supervisor and/or Human Resource department). The employer is legally required to provide “reasonable work adjustments” [12]. At 6 months, compared to the control group (usual care), the intervention group had significantly reduced RA-WIS scores, better work satisfaction, self-reported work performance and daily living function, and less pain [13]. This trial provided a higher dose of JRVR than most others [7, 9, 10].

The second UK trial (*n* = 55) was the feasibility study for this Workwell trial [14]. This used a combination of approaches from the three successful trials [9, 10, 13], whilst learning lessons from the two Dutch trials [6, 7]. People with IA were recruited, with “concerns about health affecting their ability to work in the next few years” [9] (most of whom also had a RA-WIS score ≥ 10), and participants had to be currently performing their job. (Otherwise eligible patients who were on short-term sick leave at time of screening were delayed until returned to work). The intervention was provided by work rehabilitation-trained occupational therapists, using the WES-RC, to identify work problems and solutions [14]. Individualised treatment had similar content to that provided in the Macedo et al. [13] trial, using action plans, with self-management training being work-focused and omitting the group self-management meeting (due to a lack of administrative support and viable participant numbers to organise these at trial sites). Other non-work occupational therapy interventions could be provided, if relevant. Further details of the JRVR intervention are below and in Additional file 1. At 9 months, the intervention group had improved scores in the RA-WIS, work limitations questionnaire, confidence in managing arthritis at work, physical function, pain, hand pain and perceived health status, compared to the control group (written work information) [14], and participants valued the input from therapists and were positive about the impact of JRVR on their work [15].

A further trial has since been conducted, although results are only available in abstract form [16]. This trial recruited participants (*n* = 564) from rheumatology departments and community settings across three states in Canada. It included five online work self-study modules, supported by five biweekly online group meetings led by an experienced arthritis self-management group facilitator, to enable making changes at work. This was followed by two individual meetings: a telephone consultation with a VR counsellor who recommended relevant job accommodations and provided VR counselling to support requesting these; and an in-person meeting with an occupational therapist, to identify and recommend ergonomic accommodations [17]. From 6-month through 2-year follow-up, compared to the control group receiving usual care and written work information, the intervention group had significantly improved RA-WIS scores and fewer short-term job losses [16]. This online intervention seems promising, although would best be delivered (as in the trial) at the regional or national level, as it requires greater infrastructure and administrative support, as well as patient numbers, than individual rheumatology departments can normally provide, in order to be viable and delivered regularly enough to meet patient’s needs.

The systematic review concluded that the heterogeneity of JRVR content, duration, dose, and of work outcomes used means it is difficult to identify recommendations for future trials, but that process evaluations should be nested in trials to investigate the quality of implementation, mechanisms, and contextual factors associated with variation in outcomes, with detailed descriptions of JRVR content to help establish “what works and for whom” [5]. Differences in prevailing economic conditions, social security, and health services between countries, may mean that positive results for JRVR in one country may then not translate to another country, or over time (especially in relation to economic conditions). Continued research is needed to identify if JRVR is effective and why.

The Workwell trial is a multi-centre randomised controlled trial evaluating the clinical and cost-effectiveness of Workwell JRVR. Participants are employed people with IA experiencing work instability and at risk of job loss. As described above, we tested the acceptability and utility of Workwell JRVR in a feasibility trial [14]. In the Workwell trial, JRVR is delivered by additionally trained National Health Service (NHS) occupational therapists. Outcomes are collected at baseline and 6 and 12 months post randomisation. A parallel economic evaluation is being undertaken. The Workwell trial protocol is published [18]. This paper presents the protocol for an embedded design mixed methods process evaluation alongside the Workwell trial.

### Workwell JRVR and training

Workwell JRVR uses biopsychosocial and self-management approaches and includes behavioural change techniques to facilitate people making changes in the workplace. As indicated above, the development of the Workwell JRVR content builds on three successful JRVR trials [9, 10, 13]. Workwell JRVR includes provision of a self-help written information pack of work resources (which the control group also receive). The first meeting includes a detailed work interview (Work Experience Survey – Rheumatic Conditions: WES-RC [11, 19, 20]), which uses a biopsychosocial approach to identifying work barriers. Patient and therapist collaboratively prioritise three work problem areas to address. An individualised JRVR programme is collaboratively agreed. This is provided over up to three additional treatment sessions, with a telephone review to assess overall progress and implementation of any job accommodations in the workplace. Behavioural change techniques include the following: goals and planning (goal setting, problem-solving, action planning, reviewing goals; discrepancy between current behaviour and goals); feedback and self-monitoring of behaviour; and shaping knowledge (instructions in behaviour). With the participant’s consent, employer liaison occurs to facilitate changes in the workplace, which can be in the form of a support letter specifying recommended job accommodations; a meeting (in person or remotely) with participant and manager; and/or a work site visit to further assess for ergonomic accommodations and advise in situ, if applicable (See Additional file 1). To ensure maximal accessibility to working patients with arthritis, Workwell JRVR is designed to be deliverable outside the workplace and even if participants choose not to disclose their condition at work.

Patients with symptoms of IA presenting to their General Practitioner should be referred rapidly to a rheumatology consultant [21]. If requiring continuing treatment, they then receive this from Rheumatology services in the NHS. Accordingly, these are convenient locations to deliver Workwell JRVR for people with IA, as there are early and ongoing opportunities for staff to identify JRVR needs. JRVR can also be provided in other contexts.

Occupational therapy includes VR within its scope of practice. Occupational therapy focuses on enhancing health and wellbeing through supporting participation in life roles. It uses a biopsychosocial approach with interventions focusing on the person (e.g. physical, psychological, social); their environment (e.g. physical, social, societal, cultural, economic, attitudinal), and their occupations (i.e. the activities people want to, need to, and are expected to do). The therapist helps the person bring about change to achieve their chosen goals [22].

JRVR provision in rheumatology services varies considerably. It is usually provided by occupational therapists, although can also be provided by physiotherapists. However, it is often limited to provision of written information, advice, and signposting to other services, lasting around 45 min, although some departments offer structured JRVR [23, 24]. We therefore aimed to upskill therapists in JRVR. We chose not to train whole rheumatology teams, as this could change the context in which the trial was conducted, increasing the risk of control participants receiving JRVR and contamination, as this is an individually randomised trial. We planned to recruit occupational therapists and physiotherapists, as both already have skills assessing physical and functional status in IA. Unfortunately, no physiotherapists were able to join the study, for several reasons. At the time of trial planning (2017–2018), we had greater difficulty identifying and contacting rheumatology physiotherapists, in comparison to rheumatology occupational therapists, to identify interested sites with staff capacity to support the trial. At the time, there was not yet an established national network for rheumatology physiotherapists, as there was for occupational therapists (the Royal College of Occupational Therapy Trauma and Musculoskeletal Health Specialist Section: Rheumatology Forum, with around 100 members, of which two of the authors (AH, YP) are active members already engaged in research with Forum members). This meant we had to use a snowballing approach of informal networks with rheumatology physiotherapy colleagues, primarily in the North-West and Midlands of England; asking rheumatology occupational therapists, at sites expressing interest in the trial, to liaise with physiotherapists in their NHS Trusts and local networks; and contacts with several National Institute of Health Research (NIHR) Comprehensive Research Networks (CRN: which support research infrastructure in the UK). We initially had 28 interested sites, six including physiotherapists. However, 10 sites (including all six with physiotherapists) could not continue due to either concerns from the sites’ therapy service managers about payment of trial excess treatment costs (i.e. payment for staff time to deliver treatment), as the NIHR procedure for providing such payments was changing (during 2018) and could not be confirmed; staff changes, e.g. maternity leave or other staff leaving, meant there was no longer staff capacity to take on trial work; or interested physiotherapists were unable to attend any of the Workwell training courses available. Unfortunately, it was not feasible to provide training for individual therapists.

A 2-day face-to-face Workwell training course was provided to participating occupational therapists. This was repeated three times over a 2-month period, to provide choice of attendance dates. This included how to use the WES-RC, treatment planning case studies, and practical workshops of solutions to common work problems. Following this, each therapist needed to successfully complete a mock telephone WES-RC interview, with a Workwell trainer role-playing being a patient; collaboratively identify their three key work issues; and develop a treatment plan. Therapists also had the opportunity for a formal telephone mentoring meeting with a Workwell trainer to discuss treatment plans for their first participant. Thereafter, regular mentoring sessions were offered to therapists to discuss participants’ treatment [18].

### Process evaluation

**Fig. 1:**
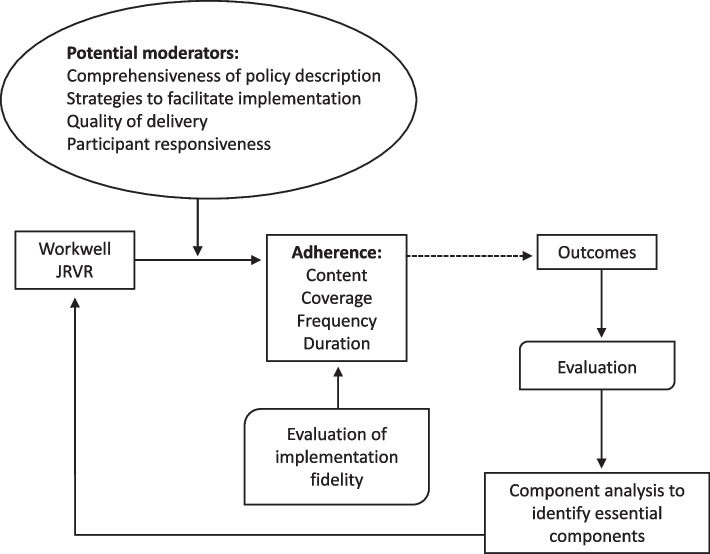
Assessment of fidelity and factors moderating Workwell JRVR delivery (CFIF)

The United Kingdom Medical Research Council (UK MRC) framework provides guidance for the process evaluation of trials with complex interventions [25]. The framework emphasises the relationships between implementation, mechanisms, and context and outlines the need for theory-driven process evaluations to measure what was delivered. A conceptual framework to assess implementation fidelity (CFIF) was proposed by Carroll et al. [26]. This includes evaluating intervention adherence (whether it was delivered by therapists as planned), including the dose, i.e. content (what was received), frequency and duration (the amount received), and coverage (whether everyone due to receive the intervention did so [26]. The degree of implementation fidelity can also be affected by moderating factors such as: intervention complexity (how detailed the intervention is); facilitation strategies to enable uniform delivery (e.g. provision of manuals, training, feedback to therapists on delivery); quality of delivery (e.g. whether behavioural change strategies are being applied as planned); and participant responsiveness (acceptance by and acceptability to those receiving it, including those receiving and providing it). From an evaluation of fidelity and moderating factors, it may be possible to identify the essential components of an intervention. Components of the CFIF in relation to Workwell JRVR are summarised in Fig. 1.

Alongside fidelity, understanding key stakeholders’ perspectives of the intervention and the social and structural context in which it is provided are essential elements. Normalisation process theory (NPT) can help in understanding how participants and therapists make changes to embed new working practices into their working lives and daily practice, respectively, as well as understanding impact on other key stakeholders [27, 28]. This includes four constructs (coherence, cognitive participation, collective action, reflexive monitoring) each informed by four components (see Table 1). NPT can be used to help synthesise data from different sources to understand contexts and mechanisms helping or hindering implementation of interventions.

**Table 1:** Normalisation process theory (adapted from May et al. 2015 [28])

NPT constructs	Components	Explanation
**Coherence**		**The sense-making work people individually and collectively do to implement changes to existing working practices. This includes:**
	Differentiation	Differentiating new practices from existing ones;
	Communal specification	Building a shared understanding of the aims, objectives, and benefits of new ways of working;
	Individual specification	Individuals understanding what they need to do
	Internalisation	Understanding the value, benefits, and importance of new ways of working
**Cognitive participation**		**The relational work people need to build and sustain new practices. This involves:**
	Initiation	Whether or not key people are driving the change forward;
	Enrolment	The work to organise/ reorganise oneself and others to collectively contribute to new ways of working
	Legitimation	Helping people believe it is right to be involved and they can actively contribute
	Activation	Once underway, collectively define actions to sustain practice and involvement
**Collective action**		**The operational work needed to implement changes in practice. This includes:**
	Interactional workability	How people interact with others/objects and key elements of new practices to put them into everyday practice
	Relational integration	How they develop the knowledge and confidence to use new practices;
	Skill set workability	The skill sets needed to do the work;
	Contextual integration	Resourcing new practices and implementing polices to enable their use
**Reflexive monitoring**		**The appraisal work to assess and understand how new practices affect them. This includes:**
	Systematisation	Collecting information to determine how effective and useful new practices are for themselves and others
	Communal appraisal	Working together to appraise the usefulness or effectiveness of changes in working practices and how these affecting existing work
	Individual appraisal	Individually appraising effects of new practices on them and work context
	Reconfiguration	How these appraisals may be leading to modifications in new practices

### Process evaluation aims/objectives

The aims of the process evaluation are to investigate fidelity to delivery of Workwell JRVR, understand the social and structural context in which the intervention is delivered, and identify factors which may influence the quality of implementation. Specific objectives include to:

1. Measure fidelity to delivering Workwell: adherence (content, coverage, frequency, and duration)
2. Assess therapists’ ability and confidence in delivering the intervention
3. Understand the content and delivery of usual care in both intervention and control groups

Investigating social and structural context will include to:

(4) Describe participating sites work services pre-Workwell trial
(5) Understand therapists’ views of the Workwell training and ways in which they consider this might be improved and delivered in future
(6) Understand therapists’ experiences of delivering the intervention
(7) Understand participants’ experiences of the intervention
(8) Understand employers’ views about their employee’s participation in the intervention
(9) Understand participants’, therapists’, therapy line managers’, and employers’ views of what social and structural factors might support implementing Workwell JRVR and
(10) Identify potential contaminants in the trial.

Data will be synthesised to gain insight into implementation, moderating factors, essential components, contexts, and mechanisms of Workwell JRVR. The synthesis will then be considered in the light of trial findings.

## Methods

### Study design

**Fig. 2:**
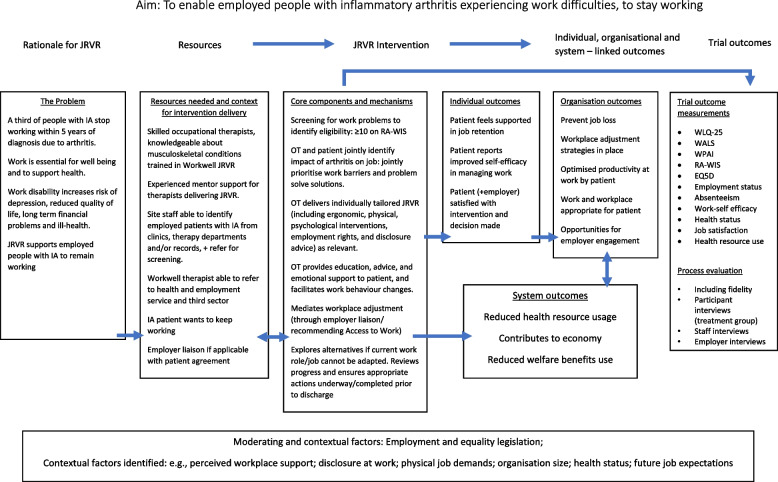
Workwell Job Retention Vocational Rehabilitation (JRVR) Logic Model

The embedded-explanatory mixed methods design [29] process evaluation will be an integral part of the Workwell trial. Investigations will draw on the Logic Model developed for the intervention (Fig. 2). We will use the CFIF to examine fidelity to Workwell [26]. Fidelity acts as a moderator between interventions and intended outcomes. Its evaluation allows for investigating whether any positive outcomes could be improved on, and whether negative outcomes are due to poor implementation or issues with the intervention. The process evaluation will also be guided by the NPT [28]. This will influence the structure of interview topic guides with participants and therapists, as well as participants’ employers and the therapy line managers, support the interpretation of the Thematic Analyses of interviews, and aid synthesis of data across data collection methods [30]. NPT facilitates understanding the perspectives of both therapists delivering JRVR in practice and participants embedding JRVR interventions into their daily lives. The process evaluation team include members of the research team who developed the Workwell JRVR, therapist training, and trial design, as well as those involved in trial management, but not analysis of the trial outcomes.

To assist future comparative evaluations of VR trials, the process evaluation protocol was planned to be similar to that within the RETAKE trial, evaluating Early Stroke Specialist Vocational Rehabilitation to enable people in returning to work [31, 32].

### Study participants

Participants include the following: trial participants in the intervention group who received Workwell JRVR, either continuing in employment or no longer employed at 12-month follow-up; trial participants in the control group; line managers or employers of participants receiving Workwell JRVR; Workwell-trained occupational therapists; and therapy line managers of participating therapists.

### Informed consent

All participants will be provided with an information sheet and opportunity to ask questions. (A copy of the trial participants’ information sheet and consent form are included in Additional files 2 and 3). Taking part in the process evaluation will be optional. Written consent to participate in semi-structured interviews, surveys, questionnaires, or focus group (as relevant) will be received from participants. This includes consent for audio-recording interviews, focus groups, and the initial JRVR treatment session for one participant each therapist treats.

### Patient and public involvement and engagement

Patient and Public Involvement and Engagement (PPIE) were ensured in all stages of the trial through the trial PPIE Group (PPIEG), of three members. A member of the PPIEG is a co-applicant on the grant and assisted in identifying research questions and designing the study and trial protocol, and is also a member of the Trial Management Group and Trial Steering Committee. All three members meet regularly and have assisted with review of patient-facing materials, including interview topic guides; advising on communication with participants; and planning and conducting the PPIEG participant interviews (see later). The PPIEG will be involved in discussion of trial results, data analysis and interpretation of process evaluation findings, and presentation of results.

### Data collection

Table 2 indicates the relationship between research aims, questions, data sources, and methods. Data sources are described below. Data collection or extraction will be conducted by members of the process evaluation team.

**Table 2:** Workwell process evaluation research questions and data sources

Aims	Research questions	Data source(s)	Method(s)
**Measure fidelity to the intervention**	What is the intervention frequency and duration? What is the intervention coverage?	• Treatment records (timing, duration of meetings, delivery method); treatment log in Workwell treatment notes • Recruitment rates; reasons for non-participation; attendance; and withdrawals	Quantitative
	What is the content of the Workwell intervention? What is the content of usual care?	• Intervention content analysis of WES-RC and Workwell treatment notes • Patient-reported resource use data • 6m questionnaire data (other work services accessed)	Quantitative and qualitative
	Was the intervention delivered with fidelity? What factors affect implementation fidelity? (context, adherence, moderating factors)	• Fidelity checklist: assessing audio recordings of WES-RC interviews; WES-RC and treatment notes; matching treatment plans to treatment note contents • Feedback to therapists on mock WES-RC performance • Mentoring records • Workwell therapist interviews • Interviews with participants (treatment as expected; satisfaction with intervention) • JRVR satisfaction item in 6-month questionnaire	Quantitative and qualitative
**Determine Workwell therapists’ ability to deliver intervention**	Are the Workwell occupational therapists confident and able to deliver the Workwell intervention?	• Therapist own assessment pre- and post-Workwell training • Individual therapist performance in mock WES-RC and treatment plan • Therapist interviews	Quantitative and qualitative
**Understand the social and structural context and identify factors which may influence intervention quality (enablers and barriers, contextual factors associated with variations in outcome across the intervention groups, factors supporting implementation into routine practice)**	What is the context for intervention delivery? Pathway for referral to occupational therapy for work advice? What services are in place for supporting patients to stay in work? What are the occupational therapy staffing levels at the site?	• Pre-trial site survey of existing work services • Therapist interviews of work service at baseline	Quantitative and qualitative
	Are there any proposed JRVR service developments or changes in practice in place/ planned at site?	• Pre-trial site survey of existing work services • Therapist interviews of work service at baseline • Therapist interviews post-Workwell provision	Quantitative and qualitative
	What are the Workwell occupational therapists’ perceptions of the training and mentoring to deliver the intervention?	• Questionnaire pre- and post-training • Therapist interviews	Quantitative and qualitative
	How do the Workwell therapists experience delivering the intervention?	• Workwell therapist interviews • Mentoring records	Qualitative
	What are the social and structural factors supporting or acting as barriers to intervention implementation?	• Workwell therapist interviews • Workwell therapist line manager interviews • Participant interviews • Participant employer interviews • Focus group with therapists • PPI group interviews with participants	Qualitative
	How do participants’ experience being supported to stay at work? How do employers view the Workwell intervention?	• Participant interviews • Participant employer interviews	Qualitative
**Identify potential contaminants**	What factors threaten the success of the trial?	• Patient-reported resource use data (changes to workplace data) • 6m questionnaire data (other work services accessed) • Therapist interviews	Quantitative and qualitative

#### Measuring fidelity

A range of predominantly quantitative data collection methods will be used informed by the CFIF (Table 3).

**Table 3:** Conceptual framework for implementation fidelity-led data extraction for fidelity assessment

Fidelity measure	CFIF construct^**a**^	Measurement tool	Data for extraction	Time point
Frequency Duration Intensity (time spent per session) Dose (number of sessions) Content (what was needed and delivered)	**Adherence and moderating factors**	**Workwell Treatment Records part 1 and 2** **WES-RC treatment notes and treatment log** **6-month questionnaire**	Intervention start date and end date Number of attended sessions Time spent (in minutes) on VR activities per session Description of problems identified, three priority problems, and interventions delivered in each session Work services received and sources	One record per participant at start (part 1), during/at discharge (or discontinuation) (part 2) Reasons for discontinuing (if applicable) One per participant: WES-RC, Treatment Notes and Treatment Log completed following each treatment session
Therapist adherence Factors affecting adherence	**Adherence and moderating factors**	**Training: Mock WES-RC** **Fidelity checklist** **Mentoring record forms**	Ability and confidence to conduct and plan Workwell Components delivered, factors affecting delivery Workwell process followed Y/N Mentor’s comments on therapists’ delivery Factors affecting intervention delivery	Therapist questionnaire pre-post training; Workwell trainer evaluation of therapist mock WES-RC ability Applied to one selected completed case per Workwell therapist Completed at formal mentoring session by Workwell mentor
Barriers and enablers to intervention delivery	**Moderating factors**	**Interviews with Workwell Therapists**	Factors affecting intervention delivery Potential solutions (developed by OT)	One Workwell therapists from each site (who delivered Workwell)
Acceptability of the intervention Barriers and enablers to intervention delivery	**Moderating factors**	**Interviews with Workwell participants, and their employers**	Acceptability of intervention Factors affecting delivery Potential solutions to barriers	Interviews with selected participants (employed; no longer employed at 12m) Interviews with participants’ line managers/employers PPIE interviews with intervention and control group participants

Key; ^a^CFIF Adherence includes intervention content, dose, coverage, frequency and duration of intervention; CFIF Moderating factors include participant responsiveness, intervention complexity, strategies to facilitate implementation, quality of delivery, recruitment, and context

##### Workwell therapists’ ability and confidence to deliver treatment

Each participating Workwell therapist will complete a questionnaire before and shortly after the training programme about their knowledge of and confidence in delivering components of Workwell JRVR, and views about delivering evidence-based practice [33]. Following therapists’ completion of the mock telephone WES-RC and treatment plan (as part of training), the Workwell training team will use a checklist to record each therapist’s ability to complete components appropriately, with feedback provided to therapists. Mentoring checklists and associated recommended action points for therapists will also be reviewed to explore therapists’ abilities when assessing and developing treatment plans for their first participant.

##### Treatment records—Workwell frequency, duration, coverage

Workwell therapists complete Treatment Records Part 1 (at start) and Part 2 (at discharge) for the trial participants they treat. These summarise if treatment started within 4 weeks; numbers of treatment sessions attended and duration; work site visit and duration (if occurred); and telephone review and duration. From this frequency, duration and time scale of treatment can be derived. Records also include if participants did not attend or were unable to attend any appointments; reasons for discontinuation (if occurred); mode of treatment delivery; any other treatment provided; participant-reported adverse events; and any participant-reported benefits of Workwell JRVR.

##### WES-RC and treatment notes—Workwell content

Treating therapists complete the WES-RC and treatment notes for each trial participant they treat. This includes the participant’s individual work barriers, three priority problems with work and JRVR solutions and resources suggested; whether there was evidence of action planning, with solutions recorded in notes as provided and implemented; a summary report letter provided to the participant (and employer letter regarding recommended job accommodations, if relevant); and duration of JRVR components (direct and indirect).

##### Intervention fidelity

A structured checklist will be used to assess fidelity of intervention delivery. Normally, treatment for the second participant of each therapist will be assessed, as this allows therapists’ time to have gained experience in Workwell JRVR delivery. Assessment will be conducted by members of the Workwell training team. Data will be obtained from the audio-recording of the participant’s initial treatment session (i.e. WES-RC interview, joint problem prioritisation, and initial treatment planning); their completed WES-RC assessment form, Workwell treatment notes, accompanying documentation (e.g. discharge letter), and Treatment Record Parts 1 and 2. The checklist will be used to identify that the Workwell process was followed: (a) the WES-RC interview was conducted appropriately; (b) from this, priority problems were appropriately established with the participant; (c) treatment content was planned that was appropriate for the agreed problem priorities of the participant; (d) treatment content was delivered as planned within an appropriate time scale, with information recorded if any issues in treatment implementation occurred (e.g. the participant chose to discontinue treatment, went on long-term sick leave, and was unable to implement); and (e) the final report for the participant reflected the treatment provided.

##### Other work provision, satisfaction with JRVR, and usual care

Additionally, in the participants’ 6-month follow-up questionnaire, we will include items to identify if intervention and control participants received: work advice, source(s) of this (written, health professional, employer-based, or other), and content. We will also identify if they report receiving and reading the work self-help information pack and their satisfaction with work advice received in the Workwell trial. Participant-reported health, social, and work resource use is also collected at 6- and 12-month follow-up.

Fidelity data will also be collected during therapist interviews (see below).

#### Social and structural context

A range of predominantly qualitative data collection methods will be used. All interviews will be conducted using a topic guide informed by NPT, except the PPIEG interview. Examples of question topics and how they relate to the four NPT constructs are shown in Additional files 4 and 5.

##### Pre-trial site survey and therapist interviews about usual VR service provision

Prior to Workwell provision, a survey will be completed by the lead therapist at each site to identify what work advice or JRVR is normally provided to patients with IA. Data collected will include the numbers of patients provided with work advice each month; average duration of advice given; and a brief description of what this consists of. Following the site training visit, the lead Workwell therapist at each site will be interviewed about their therapy and rheumatology services’ current JRVR provision. This will be a short (15 min) telephone interview, including their views at the outset on whether Workwell JRVR could be implemented in future, potential barriers, and enhancers.

##### Questionnaires and interviews with therapists about Workwell training

Each participating Workwell therapist will complete a questionnaire shortly after Workwell training asking about the relevance of each component of the training programme. Following completion of delivering Workwell JRVR at their site, therapists (one from each site *n*=18; or until data saturation is reached) will take part in a semi-structured telephone interview. This will include asking about their views of the Workwell training programme. There will also be an opportunity for therapists to take part in a focus group exploring future methods of implementing Workwell training, including using online training. This will build on work in the feasibility study, in which therapists provided views on training received and made recommendations for changes, now made in this trial [14, 34].

##### Interviews and focus groups with therapists about delivery and implementation of Workwell

The therapist semi-structured telephone interviews (see above) will then explore therapists’ views about delivering Workwell JRVR during the trial, fidelity of delivery, future implementation in practice, and whether their usual work advice service changed during the trial.

##### Therapy service managers’ semi-structured interviews

Therapists at each site will identify their appropriate therapy line manager to be contacted for consent for a 10- to 15-min telephone interview (one from each site *n*=18, or until data saturation is reached). Managers’ views will be explored about Workwell JRVR implementation during the trial, and potential future implementation in clinical services.

##### Interviews with trial participants

Semi-structured interviews will explore trial participants’ views about Workwell JRVR. This will focus on identifying which components of the Workwell intervention participants implemented, whether/ how their job changed as a result of the Workwell intervention, which components they consider most or least helpful to assist them staying in work (e.g. job modifications, flexible hours, self-management), whether JRVR enabled them to make changes (if any), and what contextual factors helped them to stay in work. If participants are no longer working, contextual factors contributing to them stopping work and their views of the JRVR received will be explored. Trial participants’ acceptability of the intervention and its provision within the NHS will also be explored. We will also similarly explore control group participants views of the written information pack provided.

One-to-one telephone interviews will be sequentially completed after participants have completed their 12-month follow-up questionnaire. This will ensure that they have completed JRVR, and sufficient time has passed for any changes to have an effect (if any) on their work. Interviews will be undertaken with purposefully selected participants in the intervention and control groups who consent to being contacted for interview. Participants will be selected based on those who are in work (up to *n*=15) and not in work (if any, up to *n*=10) in each group, reflecting the gender distribution of IA (two-thirds women) and across four job skill level groups, with three to four each from Level 1 (elementary occupations); Level 2 (administrative, caring, leisure, sales, customer service; process, plant, and machinery operatives); Level 3 (associated professional and technical/ skilled trades); and Level 4 (professional and managerial) [35]. Participants’ job skill level groups are identified from their job titles in their baseline questionnaires. Interviews will usually last 45 to 60 min.

Additionally, members of the PPIEG will also conduct semi-structured telephone interviews (10 to 15 min) with a convenience sample of participants from both the intervention and usual care arms of the trial, who consent to being contacted for interview after the 12-month follow-up questionnaire is completed. The interview topic guide was developed by the PPIEG and differs from above as it focuses on trial procedures, the patient-facing documentation, and trial participants’ views implementation of Workwell JRVR and the self-help information pack in the NHS.

##### Interviews or surveys with participants’ line manager/ employer

A selection of intervention group participants’ line manager or employer (*n*=10) will be interviewed or surveyed about their views of the Workwell JRVR received by their employee. Following completing the 12-month follow-up questionnaire, participants will be provided information about the line manager/ employer interview or survey to consider. For those interested, they are asked to discuss the study with their line manager/employer. They will be provided with a coaching script as a guide to help them explain this part of the study (if they wish to use it). If their line manager/employer agrees to take part, a member of the process evaluation team will receive their documented verbal consent at the agreed time/date of their interview and then conduct the telephone semi-structured interview (10 min). We also have the option for line managers/ employers to complete a short survey by email, if they do not have time to, or prefer not to, take part in an interview. Any surveys returned can be considered as providing consent. It is highly likely that participants who have already disclosed their arthritis to their line manager/employer are more likely to agree to employer contact. For those participants not interested in employer contact, we will ask if they are willing to indicate why not. We will explain there are many reasons why people prefer not to have this contact and that their response will help us to understand these.

##### Identifying potential contaminants

During therapist interviews, we will ask if participants in either group received more work-related intervention than planned in the trial (see Additional file 5). Additionally, we will use 6-month questionnaire data (about other work services accessed) and patient-reported resource use data (changes to workplace data) to identify whether participants in the treatment or control groups received work interventions from elsewhere, and whether this differed between groups.

### Data analysis

The process evaluation team will analyse data collected and support the PPIEG in analysing the interviews they conduct, if required. Quantitative data will mainly be analysed and presented using descriptive statistics. Interviews and focus groups will be digitally audio-recorded and transcribed verbatim, with names replaced by codes or pseudonyms. Qualitative data will be managed using the NVivo 12 software. Qualitative analysis will be done inductively based on Braun and Clarke’s thematic analysis method [30]. Then, the codes will be organised into themes and mapped under the four NPT constructs. Pseudonyms will be used where participants’ accounts are directly quoted.

To ensure internal validity and reliability*,* the following strategies will be employed: each transcript will be checked against the recording to ensure no mistakes during transcription; validity and reliability of the emerging themes will be supported by asking two researchers to analyse the data independently and agree themes; one other member of the team will then independently review two interview transcripts with participants and two with therapists and their analyses [36]; and through regular discussion of the themes and definitions with other members of the process evaluation team and PPIEG. The final relevant components of the report (e.g. participant interview report to participants) will be returned to interviewees to confirm it reflects their experiences [30, 36].

Workwell JRVR adherence will be calculated using data from the participants’ Treatment Records completed by Workwell therapists. Descriptive data will be extracted on frequency, duration, intensity, and dose of JRVR. If Workwell was not attended or discontinued, reasons will be extracted if recorded. To identify Workwell content, the following will be analysed: WES-RC, treatment notes, summary discharge report to participant, any other documents provided (e.g. other communications with participants, employer letter) and Treatment Log (i.e. coded content of the treatment provided, identifying what types of VR (direct and indirect) were provided, e.g. conducting the WES-RC interview, providing VR, using resources to identify solutions, writing reports). The numbers of work site visits and employer contacts will also be recorded. From the WES-RC, we will descriptively analyse frequency of health issues reported as affecting work (e.g. pain, fatigue, stress); type of work (categorised into job skills levels 1 and 2, or 3 and 4); work barriers identified, and priority problems identified. The WES-RC therapist notes will be content analysed to identify the VR solutions provided and whether solutions were reported by patients as implemented [37]. This data will be mapped on to the Template for Intervention Description and Replication framework (TIDieR) to describe the Workwell intervention delivered [38]. We will also use TIDieR to analyse data collected from the 6-month follow-up questionnaire to describe usual care received by both intervention and control groups.

The audio-recording of each therapist’s initial treatment session with one participant (up to *n*= 35) will be listened to by members of the research team, using a structured fidelity checklist, to assess whether the therapist went through the assessment process appropriately, identified and appropriately prioritised the participant’s problems, planned, and commenced an appropriate treatment plan. The completed WES-RC and the accompanying trial treatment notes for that participant will also be analysed to evaluate whether the problems, plan, and solutions, as discussed, were recorded in the WES-RC and the treatment notes record the fulfillment of the treatment plan [39–41].

Therapist training questionnaire data will be analysed descriptively using medians (IQRs) to explore views about components of the training. Inferential statistics will be used to investigate if there is any self-perceived change pre- to post-training in knowledge of and confidence in delivering Workwell JRVR and adopting new interventions into practice. We will also content analyse comments in the post-training questionnaire made about the training duration and content, and the mock telephone WES-RC interview and mentoring checklists, to further understand therapists’ views about training and ability to deliver Workwell.

### Data synthesis

The analysis of the different elements of the process evaluation will be conducted prior to the main trial findings being available. Each component will be reported separately. The process evaluation team will then review and integrate the components. Investigation of context, implementation, and mechanisms of impact will be guided by both CFIF [26] and NPT [27], with reference to the Workwell logic model. NPT provides structure to process evaluations by focusing attention on the range of actors, times, and places involved in implementing interventions [28]. It will also aid data synthesis from multiple sources (participants, participants’ line managers/ employers, therapists, service managers) and will provide a framework to assist understanding the mechanisms of actions of JRVR, if the trial is successful. Emerging findings from the different components of the process evaluation will be discussed amongst the team and with the wider trial team to facilitate transparency.

The process evaluation data will be analysed independently from the Workwell trial outcome evaluation. The analyses are conducted by two separate teams. Once the process evaluation and Workwell trial data analyses are complete, the process evaluation findings will be reviewed in the light of trial findings. Reviewing both analyses can aid understanding why the intervention or different components were successfully implemented or not, potential mechanisms of impact and explain trial outcomes. We will discuss the findings with therapists to further explore issues around service implementation, including identifying people with JRVR needs and methods of delivering training. The findings from the trial participant and PPIEG interviews will especially aid us in understanding what working people with arthritis consider applicable ways to implement JRVR.

The Workwell trial has been impacted by the COVID-19 pandemic. A third of the way through the treatment period, the trial had to pause for between 4 and 8 months across different sites. At re-start, therapists switched to remote Workwell JRVR delivery via telephone or videoconferencing, whilst working in very stretched circumstances. Many participants were experiencing increased job, personal and health stresses, working from home, and unused to remote treatment. We will also therefore explore participants’ and therapists’ views of face-to-face versus remote JRVR delivery, within the context of these difficult circumstances.

## Discussion

Process evaluations are recommended to be embedded into randomised controlled trials of complex interventions [25]. However, there is no single way to conduct a process evaluation [41]. Process evaluations cannot provide answers to all aspects of complex interventions [25]. Practical limitations of time, resources, and staffing can restrict the focus. We have therefore prioritised what is achievable within limited resources. To our knowledge, one other JRVR trial in arthritis has conducted a process evaluation [41]. This focused on implementation (recruitment, reach, dose, treatment fidelity, and satisfaction). The Workwell process evaluation focuses on examining fidelity, as well as understanding key components of the social and structural context from participants’ and therapists’ perspectives. This will help us to understand: what is in the “black box” of this complex intervention and frequency of the JRVR components delivered; how therapists are tailoring JRVR to individual needs and how they do this within their clinical contexts; and how participants experience JRVR, what mechanisms they consider can change and what JRVR components they are able to implement in their working lives. Process evaluations can also help research teams in interpreting how study contexts and mechanisms contribute to outcomes, as well as exploring issues for post-trial implementation, if successful [42, 43].

We have included interviews and surveys with employers as key stakeholders, notably participants’ employers’ or line managers. Employers should be considered as part of the team enabling the person to continue to work [44]. However, we anticipate it may be difficult to interview employers/ line managers. In our feasibility study, we were unable to interview any [14]. Participants who have not disclosed their condition at work, or with unsupportive managers not enabling reasonable adjustments, are unlikely to facilitate contact with their employer/ line manager. Participants may also make “informal” modifications, only providing limited explanations of these to their line managers, and so not perceive it relevant for their line manager to be interviewed. Accordingly, we consider we may only gain limited insights into Workwell JRVR impacts on employers and workplaces.

We are focusing on interviewing therapy line managers to begin exploring implementation in clinical services. Arguably, we should also include interviewing rheumatology teams. This would help understand their views on feasibility of identifying patients needing JRVR, as well as integrating JRVR into rheumatology services. The trial recruited participants from rheumatology and therapy departments using the RA-Work Instability Scale [8]. A score of 10 or more indicates the need for JRVR and potential risk of future job loss. Strategies to implement this in practice could be explored in future, e.g. using the rheumatology ePROMS system being rolled out [45]. We will explore this in future if time allows.

There are high levels of job loss amongst people with IA. Unemployment in people with chronic diseases is known to be associated with poorer physical and mental health outcomes, and reduced income and pension in retirement. As well as this personal toll, there are also increased health care and social security costs to society [46]. Effective JRVR has the potential to reduce costs to the individual, health care system, society, and employers. Most job accommodations cost employers either nothing or are relatively cheap to implement (averaging £450/person), saving employers the costs of recruiting and training replacement staff [47]. In the UK, a particular difficulty for many employed people with IA (and other long-term conditions) and their employers is accessing work advice and JRVR, as services have been described as “deficient” [48]. Whilst large organisations usually provide varying degrees of occupational health support, this is not available in most small and medium enterprises, which employ around 60% of the UK workforce. Assessments for job accommodations, and (some) financial support with costs, are available via the UK Government-funded Access to Work (AtW) scheme [49]. This primarily focuses on job accommodations and does not include the broader components of work rehabilitation provided in this JRVR programme. However, a UK survey identified two-thirds of working people with arthritis had not heard of the AtW scheme [50]. As people with IA are in regular contact with their rheumatology department, this provides an avenue for timely identification of work problems, providing JRVR and enabling access to services such as AtW.

By examining what and how JRVR strategies are implemented, what facilitators and barriers there are to doing so, and synthesising these with the trial results, this process evaluation can contribute to understanding why this JRVR programme is either effective or ineffective. This will further aid employed people with IA, health and occupational health professionals, and employers in understanding what, when, how, and with whom to implement JRVR. This JRVR could be implemented in other settings outside the health service, if those delivering it are appropriately trained or experienced. This protocol offers a blueprint (underpinned by theoretical models) for conducting process evaluations in other JRVR trials in IA and other long-term physical conditions, to help identify how trial methodology can be improved to maximise positive outcomes and minimise the risk of poor implementation.

## Conclusion

This process evaluation aims to provide insights into understanding the findings of the Workwell trial, as well as contributing to how it could be implemented into practice. This article also provides an example of how the CFIF and NPT can be included into future process evaluations of work interventions for people with arthritis, as well as other long-term conditions, who could benefit from JRVR.

## Trial status

The Workwell trial is in progress. Workwell protocol v4 04.10.2021. Recruitment was completed 31.01.2021, with final randomisations completed on 28.2.2021. Twelve-month follow-up will be completed by the 31.03.2022. Process evaluation data collection will be completed by 31.07.2022. We were unable to complete and submit this process evaluation protocol prior to recruitment being completed due to the impact of the COVID-19 pandemic on the trial. Trial pause and re-start, plus research team members having to take over recruitment and treatment-related functions (due to NHS staff redeployment), delayed production of this protocol.

## Supplementary Information

**Additional file 1.** Workwell intervention.

**Additional file 2.** Trial participants’ information sheet.

**Additional file 3.** Trial participants’ consent form.

**Additional file 4.** Workwell trial: NPT constructs mapped to participants and employer topic guide.

**Additional file 5.** Workwell trial: NPT constructs mapped to therapist topic guide.

## Data Availability

The de-identified datasets generated and/or analysed during the current study will not be publicly available (as specific consent to place data in a public database was not obtained) but will be available from the corresponding author on reasonable request. The UK WES-RC and UK WES-RC Manual are available in the University of Salford Institutional Repository (see reference list).

## Abbreviations

AtW: Access to Work
CFIF: Conceptual framework to assess implementation fidelity
CRN: Comprehensive Research Network
IA: Inflammatory arthritis
JRVR: Job retention vocational rehabilitation
MRC: Medical Research Council
NHS: National Health Service
NIHR: National Institute of Health Research
NPT: Normalisation process theory
OA: Osteoarthritis
PPIE: Patient and public involvement and engagement
PPIEG: Patient and public involvement and engagement group
PsA: Psoriatic arthritis
RA: Rheumatoid arthritis
RA-WIS: Rheumatoid Arthritis – Work Instability Scale
RETAKE: RETurn to work After stroKE
TIDieR: Template for Intervention Description and Replication framework
UK: United Kingdom
US: United States
VR: Vocational rehabilitation
WES-RC: Work Experience Survey-Rheumatic Conditions

## References

[1] Burton W, Morrison A, Maclean R, Ruderman E (2006). Systematic review of studies of productivity loss due to rheumatoid arthritis. Occup Med (Lond).

[2] Gwinnutt JM, Leggett S, Lunt M, Barton A, Hyrich KL, Walker-Bone K (2020). Predictors of presenteeism, absenteeism and job loss in patients commencing methotrexate or biologic therapy for rheumatoid arthritis. Rheumatology (Oxford).

[3] Kim D, Kaneko Y, Takeuchi T (2017). Importance of obtaining remission for work productivity and activity of patients with rheumatoid arthritis. J Rheumatol.

[4] Hoving JL, Lacaille D, Urquhart DM, Hannu TJ, Sluiter JK, Frings-Dresen MHW. Non-pharmacological interventions for preventing job loss in workers with inflammatory arthritis. Cochrane Database Syst Rev. 2014;(11): Art. No.: CD010208. 10.1002/14651858. 10.1002/14651858.CD010208.pub2 PMC11287239 25375291

[5] Madsen CMT, Bisgaard SK, Primdahl J, Christensen JR, von Bülow C (2021). A systematic review of job loss prevention interventions for persons with inflammatory arthritis. J Occ Rehab.

[6] de Buck PD, le Cessie S, van den Hout WB, Peeters AJ, Ronday HK, Westedt ML (2005). Randomized comparison of a multidisciplinary job-retention vocational rehabilitation program with usual outpatient care in patients with chronic arthritis at risk for job loss. Arthritis Rheum.

[7] van Vilsteren M, Boot CRL, Twisk JWR, Steenbeck R, Voskuyl AE, van Schaardenburg D, Anema JR (2017). One year effects of a workplace integrated care intervention for workers with rheumatoid arthritis: results of a randomsied controlled trial. J Occup Rehabil.

[8] Gilworth G, Chamberlain MA, Harvey A, Woodhouse A, Smith J, Smyth MG (2003). Development of a work instability scale for rheumatoid arthritis. Arthritis Rheum.

[9] Allaire SH, Li W, LaValley MP (2003). Reduction of job loss in persons with rheumatic diseases receiving vocational rehabilitation: a randomized controlled trial. Arthritis Rheum.

[10] Keysor JJ, LaValley M, Brown C, Felson DT, AlHeresh RA, Vaughn MW, Yood R, Reed JI, Allaire SJ (2018). Efficacy of a work disability prevention program for people with rheumatic and musculoskeletal conditions: a single-blind, parallel-arm randomised controlled trial. Arth Care Res.

[11] Allaire S, Keysor JJ (2009). Development of a structured interview tool to help patients identify and solve rheumatic condition-related work barriers. Arthritis Rheum.

[12] Gov. UK. Equality Act 2010: a summary guide to your rights. https://assets.publishing.service.gov.uk/government/uploads/system/uploads/attachment_data/file/85017/individual-rights1.pdf. Accessed 8 Oct 2022.

[13] Macedo AM, Oakley SP, Panayi GS, Kirkham BW (2009). Functional and work outcomes improve in patients with rheumatoid arthritis who receive targeted, comprehensive occupational therapy. Arthritis Rheum.

[14] Hammond A, O’Brien R, Woodbridge S, Bradshaw L, Prior Y, Radford K (2017). Job retention vocational rehabilitation for employed people with inflammatory arthritis (WORK-IA): a feasibility randomized controlled trial. BMC Musculoskelet Disord.

[15] Prior Y, Amanna AE, Bodell SJ, Hammond A (2017). A qualitative evaluation of occupational therapy-led work rehabilitation for people with inflammatory arthritis: participants’ views. Br J Occup Ther.

[16] Luqini A, Zheng Y, Xie H , Backman C , Rogers P, Kwok A, Knight A , Gignac M, Mosher D, Li L , Esdaile J, Thorne C, Lacaille D. Effectiveness of the Making It Work ^TM^ Program at improving presenteeism and work cessation in workers with inflammatory arthritis - results of a randomised controlled trial. 10.1136/annrheumdis-2020-eular.2383.

[17] Carruthers EC, Rogers P, Backman CL (2014). “Employment and arthritis: making it work” a randomized controlled trial evaluating an online program to help people with inflammatory arthritis maintain employment (study protocol). BMC Med Inform Decis Mak.

[18] Hammond A, Sutton C, Cotterill S, Woodbridge S, O’Brien R, Radford K (2020). The effect on work presenteeism of job retention vocational rehabilitation compared to a written self-help work advice pack for employed people with inflammatory arthritis: protocol for a multi-centre randomised controlled trial (the WORKWELL trial). BMC Musculoskelet Disord.

[19] Hammond A, Woodbridge S, O’Brien R, Grant M (2013). The UK Work Experience Survey for persons with Rheumatic Conditions (UK WES-RC).

[20] Hammond A, Woodbridge S, O’Brien R, Grant M (2013). The UK Work Experience Survey for persons with Rheumatic Conditions (UK WES-RC) Manual version 2.

[21] British Society for Rheumatology (BSR) (2021). Adult rheumatology referral guidelines.

[22] Royal College of Occupational Therapists (2021). Professional standards for occupational therapy practice, conduct and ethics. Version 2.

[23] Prior Y, Hammond A (2014). OP0084-HPR Do occupational therapy services fulfil the work related needs of rheumatology patients in the UK?. Ann Rheum Dis.

[24] O’Brien R, Woodbridge S, Hammond A, Adkin J, Culley J (2013). The development and evaluation of a vocational rehabilitation training programme for rheumatology occupational therapists. Musculoskeletal Care.

[25] Moore GF, Audrey S, Barker M, Bond L, Bonell C, Hardeman W (2015). Process evaluation of complex interventions: Medical Research Council guidance. BMJ.

[26] Carroll C, Patterson M, Wood S, Booth A, Rick J, Balain S (2007). A conceptual framework for implementation fidelity. Implement Sci.

[27] May C, Finch T (2009). Implementing, embedding, and integrating practices: an outline of normalization process theory. Sociology.

[28] May C, Rapley Y, Mair F, Treweek S, Murray E, Bellini L (2015). Normalization Process Theory On-Line User’s Manual, Toolkit and NoMAD instrument.

[29] Cresswell J, Plano Clark V (2011). Designing and conducting mixed methods research.

[30] Braun V, Clarke V (2006). Using thematic analysis in psychology. Qual Res Psychol.

[31] Radford KA, Craven K, McLellan V, Sach TH, Brindle R, Holloway I (2020). An individually randomised controlled multi-centre pragmatic trial with embedded economic and process evaluations of early vocational rehabilitation compared with usual care for stroke survivors: study protocol for the RETurn to work After stroKE (RETAKE) trial. Trials.

[32] Radford KA, McKevitt C, Clarke S, Powers K, Phillips J, Craven K (2022). RETurn to work After stroke (RETAKE) trial: protocol for a mixed methods process evaluation using normalization process theory. BMJ Open.

[33] Aarons GA (2004). Mental health provider attitudes toward adoption of evidence-based practice: the Evidence-Based Practice Attitude Scale (EBPAS). Ment Health Serv Res.

[34] Prior Y, Amanna EA, Bodell SJ, Hammond A (2015). A qualitative evaluation of occupational therapy-led work rehabilitation for people with inflammatory arthritis: Perspectives of therapists and their line managers. Br J Occup Ther.

[35] Office for National Statistics. Standard occupational classification 2010. https://www.ons.gov.uk/methodology/classificationsandstandards/standardoccupationalclassificationsoc/soc2010. Accessed 8 Feb 2022.

[36] Creswell J, Creswell D (2018). Research design: qualitative, quantitative, and mixed methods approaches.

[37] Bengtsson M (2016). How to plan and perform a qualitative study using content analysis. NursingPlus Open.

[38] Hoffmann TC, Glasziou PP, Boutron I, Milne R, Perera R, Moher D (2014). Better reporting of interventions: template for intervention description and replication (TIDieR) checklist and guide. BMJ.

[39] Toomey E, Matthews J, Guerin S, Hurley DA (2016). Development of a feasible implementation fidelity protocol within a complex physical therapy-led self-management intervention. Phys Ther.

[40] Toomey E, Matthews J, Hurley DA (2017). Using mixed methods to assess fidelity of delivery and its influencing factors in a complex self-management intervention for people with osteoarthritis and low back pain. BMJ Open.

[41] van Vilsteren M, Boot CR, Voskuyl AE, Steenbeek R, van Schaardenburg D, Anema JR (2016). Process evaluation of a workplace integrated care intervention for workers with rheumatoid arthritis. J Occup Rehabil.

[42] Grant A, Treweek S, Dreischulte T, Foy R, Guthrie B (2013). Process evaluations for cluster-randomised trials of complex interventions: a proposed framework for design and reporting. Trials.

[43] Kelly M, Steed L, Sohanpal R, Pinnock H, Barradell A, Dibao-Dina C (2021). The TANDEM trial: protocol for the process evaluation of a randomised trial of a complex intervention for anxiety and/or depression in people living with chronic obstructive pulmonary disease (COPD). Trials.

[44] Wilkie R, Bjork M, Costa-Black KM, Parker M, Pransky G (2020). Managing work participation for people with rheumatic and musculoskeletal diseases. Best Pract Res Clin Rheumatol.

[45] British Society for Rheumatology (BSR). BSR ePROMs: British Society for Rheumatology (BSR). Available from: https://bsreproms.org.uk/pages/home. Accessed 18 Feb 2022.

[46] Waddell G, Burton K, Kendall NAS (2008). Vocational Rehabilitation: what works for whom and when?.

[47] Job Accommodation Network. Costs and benefits of accommodations. 2020. https://askjan.org/topics/costs.cfm. Accessed 8 Oct 2022.

[48] Vocational Rehabilitation Association. 2020. https://vrassociationuk.com/about/. Accessed 8 Oct 2022.

[49] Gov. UK. Department of Work and Pensions. Access to Work: factsheet for customers. 2022. https://www.gov.uk/government/publications/access-to-work-factsheet/access-to-work-factsheet-for-customers. Accessed 8 Oct 2022.

[50] Versus Arthritis. Working it out: awareess of access to work and employer support. https://www.versusarthritis.org/media/13469/working-it-out-report_awareness-and-employer-support.pdf. Accessed 8 Oct 2022.

