# Supplementary material for: The Workwell trial: protocol for the process evaluation of a randomised controlled trial of job retention vocational rehabilitation for employed people with inflammatory arthritis
Source: Trials. 2022 Nov 9;23:937. doi: 10.1186/s13063-022-06871-z (PMC9645762; doi:10.1186/s13063-022-06871-z)
Supplement: Supplementary file 1 — Additional file 1. Workwell intervention. [file 13063_2022_6871_MOESM1_ESM.docx]

**Additional File 1: Workwell Intervention: Process evaluation protocol. Hammond et al, TRIALS 2022.**


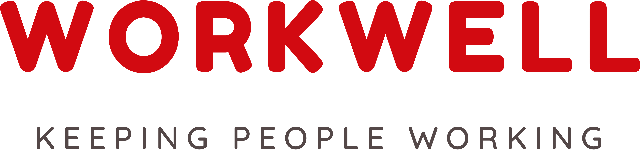


**WORKWELL Trial Process Evaluation Protocol:**

1. **Work Self-help Information Pack (intervention and control group)**

This is received by all participants. The control group only receive this. This includes:

- a welcome letter, encouraging people to read the enclosed booklets, identify and discuss work problems and solutions with relatives, friends, and employers/line managers. For the intervention group, this additionally explained the Workwell therapist would help them with this process.
- a self-help flowchart, suggesting how to identify problems, resulting work difficulties and determine solutions to then implement and/or discuss with employers/ line managers
- and four work self-help booklets including: a summary of the Equality Act [UK Government Equalities Office, 2010 ]; how arthritis can affect work, benefits of staying in work, case studies of work issues people with arthritis have and how they resolved these (providing narratives and opportunities for modelling), common work problems and solutions, staying active, disclosure, the importance of discussing work issues with employers and colleagues, what “reasonable adjustments” employers could make, and how to get further help to manage at work [“I want to Work” National Rheumatoid Arthritis Society, 2018; “Working with Arthritis” Arthritis Care 2016/Versus Arthritis; “Work and Your Wellbeing” Versus Arthritis 2018].

1. **WORKWELL Job Retention Vocational Rehabilitation (intervention group)**

WORKWELL is based on that provided in the WORK-IA trial [14] and trials conducted by Allaire et al [9] and Macedo et al [13]. It is based on the biopsychosocial model and self-management approaches. In the appointment letter in advance of the first meeting, it is emphasised that the more effort the person can put into making changes at work, and to self-manage the effects of their arthritis, the more quickly results will be achieved; and asking participants to start reading the information pack sent to them. The intervention usually consists of between two to four meetings (with the first being a work-related interview), with each meeting generally lasting up to an hour. The first is held within four weeks of the participant receiving the self-help pack. Meetings are held at around four-week intervals (or as agreed with the participant), allowing time between meetings for participant and therapist to take appropriate actions. The number of meetings held depends on the nature of the work problems identified. Participants can be offered meetings earlier and later in the day to reduce impact on the working day. A telephone review is conducted six weeks after the last intervention meeting to check progress and if job accommodations have been implemented in the workplace (or in progress).

At the first meeting, the therapist uses the UK Work Experience Survey- Rheumatic Conditions (UK WES-RC) [19; 20; developed from the US WES-RC [11]. The WES-RC uses a biopsychosocial approach and includes a wide range of topics including: getting ready for and travel to/from work; workplace access; physical, mental and time job demands; relationships with people at work; environmental factors; company policies; job satisfaction and work life balance [11, 19, 20]. The therapist works with the participant to:

- Identify work problems and specific barriers (physical, psychological, environmental (physical/social) and managerial) to overcome.
- Collaboratively agree three broad priority areas of work problems (e.g. workstation modification and work positioning; moving to/around the workplace), specifying problems within these

The therapist then recommends any applicable initial self-management advice tailored to the participant’s needs, e.g., hand exercises, relaxation, sleep hygiene, fatigue and/or joint protection/ergonomic advice and provides appropriate booklets, e.g., Versus Arthritis’ publications*: “Keep Moving,” “Fatigue and Arthritis” and “Looking After Your Joints when you have arthritis,” and/ or online resources, as applicable.

The therapist explains the benefits of action plans, and helps the participant to complete an Action Plan, with a few initial, achievable SMART goals, to meet some immediate identified needs, and help the participant start taking appropriate actions to resolve work problems. Action plans can contribute to building self-efficacy. This meeting can take 1 to 1.5 hours, depending on the degree of difficulties the person is experiencing.

Participants are asked, before the next meeting, to complete an Activity Diary, for 24 hours on a typical workday, identifying for each 30-minute period: their main activity; any equipment, tools or materials used; any difficulties or discomfort doing the activity; whether they took a short rest; and to rate their level of pain and fatigue using a 0 (no) to 10 (severe) scale [modified with permission. This was modified with permission for the UK from the Work Activity Diary [Backman C, Village L, Lacaille D, 2008]. Participants are asked to reflect on problems identified in the WES-RC and activity diary and consider possible solutions, supported by reading information in the self-help pack.

Between the first and second meeting, the therapist reviews the three priority problem areas identified in the WES-RC and develops a range of possible solutions to discuss with the participant in meeting 2. Developing solutions is supported by: using the Workwell Solutions Manual, which includes potential solutions and information resources linked to each section of the WES-RC; the therapists JRVR training and occupational therapy experience.

At this second meeting, the participant and therapist: review the Activity Diary to gain a better understanding of the person’s workday; agree which solutions are workable; and the participant is supported to progressively make changes and put solutions into place. At the start of this, and any subsequent meetings, the therapist and participant review progress with action plans set in the previous meeting, collaboratively problem-solve, and the therapist continues supporting participants to write Action Plans and in resolving difficulties for themselves. Throughout the meetings, therapists emphasise the importance of participants taking responsibility for actions and to liaise with employers to request and obtain job accommodations.

A Treatment Standard Operating Procedure was provided to all therapists to remind them of treatment procedures.

WORKWELL JRVR is individualised to address the three problem work priority areas jointly identified and the content can include any of the following strategies, as applicable and agreed with the participant:

1. applying ergonomic (e.g., altering work actions and work positioning, planning, prioritising, and scheduling), fatigue (e.g., pacing, microbreaks) and stress (e.g., relaxation, mindfulness) management approaches to the workplace.
2. recommendations for assistive technology/equipment adaptation, workplace/workstation modification, transport advice
3. practical advice and support enabling participants to: discuss the pros and cons of disclosure and how to disclose their condition at work (if not already done so); and negotiate job modifications with employers/ line managers. This can include discussion of strategies, how to explain their condition’s impact on work, and proactively make suggestions for addressing issues and role play, when applicable. Disclosure at work is an essential precursor for negotiating employer liaison.
4. explaining rights under the Equality Act 2010, how and why their condition meets being one applicable under the Equality Act, what are the range of “reasonable adjustments” they could request and employers can make in the workplace. The facilities available to support employers making "reasonable adjustments," e.g. Access to Work [<https://www.gov.uk/access-to-work>]; and how the participant can apply to Access to Work.
5. physical interventions, e.g., hand and upper limb exercise training, manual handling advice and training
6. psychological support, through listening to and discussing work problems; encouraging ability and confidence in solving work problems, managing arthritis when working, and continuing working in future
7. advice on other activities of daily living difficulties affecting work ability
8. discussing relationships with managers and co-workers, and strategies for how to improve or manage these if presenting a barrier
9. discussing work-life balance and strategies to improve this and avoid role overload
10. to discuss, if necessary, about considering a change in job, role or career. Referral to a Disability Employment Advisor (DEA) at their local Jobcentre for advice and support in doing so, if the participant wishes
11. signposting and/ or referral, as relevant, to any other services identified as beneficial within and outside of the rheumatology or community multi-disciplinary team, such as ‘mainstream' occupational therapy (e.g. for splinting, activities of daily living training, self-management education), physiotherapy (e.g. for pain management, exercise therapy), podiatry (e.g. for insole or footwear provision), rheumatology nurse (e.g. advice on managing medications); state, employer and third sector work rehabilitation services as relevant (e.g. DEA; advice to contact their employer’s occupational health department; how to contact Access to Work); and to social work, local authority or community advisory services (e.g., Citizen’s Advice Bureau) for support related to housing, financial or welfare needs.
12. provision of relevant work and self-management advice booklets, on-line resources, and other information as appropriate
13. general advice on disease flare management and contacting the Rheumatology department quickly in a flare, for review of medication changes or steroid injection, if necessary, to reduce the risk of longer sickness absences
14. optional worksite visit: to conduct a workplace job assessment, and employer/ line manager liaison if required. The participant provides written consent to the work site visit and obtains permission in advance from the employer for this to occur. The therapist, as necessary, liaises in advance with the workplace to ensure any health and safety issues attending the worksite are explained and appropriate arrangements made. Practical solutions can be recommended and instituted during the visit.
15. Employer liaison: The extent of employer liaison is guided by the participant, as the therapist is working on behalf of the person rather than their employer. A letter can be provided for the employer/ line manager about the reasonable adjustments the participant needs. The content of the letter is agreed with the participant. This can be supplemented with information for employers (NRAS: An Employer’s Guide to Rheumatoid Arthritis: <https://nras.org.uk/product/an-employers-guide-to-rheumatoid-arthritis/>). The participant can provide the letter and/or booklet to their manager to support discussing their job accommodations needs in the workplace. If the participant wishes, a meeting can also be held between the therapist, participant, and their line manager to discuss relevant job accommodations, what is feasible and about referral to Access to Work for funding for equipment and/or other support, as applicable. The content of this meeting is discussed and agreed with the participant in advance. Alternately, employer liaison may be by telephone, with the participant’s involvement.
16. A telephone review, six weeks after the final meeting, to discuss participants’ progress implementing changes, check recommended job accommodations ae in place and if any further action is needed. This lasts up to 30 minutes.
17. The participant is provided with a discharge letter, summarising the issues, actions and recommendations provided in WORKWELL. This can be written to be suitable to provide to the employer/ line manager, if the participant wishes this.

***Versus Arthritis downloads available from:**

Keep Moving:

<https://www.versusarthritis.org/media/1310/keep-moving-information-booklet-with-poster.pdf>

Looking After Your Joints:

<https://www.versusarthritis.org/media/1271/looking-after-your-joints-information-booklet.pdf>

Fatigue and Arthritis:

<https://www.versusarthritis.org/media/1269/fatigue-and-arthritis-information-booklet.pdf>

**Self-help information pack downloads:**

UK Government Equalities Office, 2010. Equality Act 2010: What you need to know. A summary guide to your rights. <https://www.equalityadvisoryservice.com/ci/fattach/get/590/1354033733/redirect/1/session/L2F2LzEvdGltZS8xNjY3NTg0NzMzL3NpZC9mbG5Vam1UcA==/filename/individual-rights1.pdf>

National Rheumatoid Arthritis Society. 2018. I want to work. <https://nras.org.uk/wp-content/uploads/sites/2/woocommerce_uploads/2020/12/134579-I-Want-to-Work-Booklet-FINAL-2021-ukmknu.pdf>

Arthritis Care 2016/Versus Arthritis. 2016.Working with Arthritis” Arthritis Care 2016/Versus Arthritis. <https://www.tims.nhs.uk/wp-content/uploads/2021/12/working-with-arthritis-booklet.pdf>

Versus Arthritis. How can I get the right support? <https://www.versusarthritis.org/about-arthritis/living-with-arthritis/work/>
