# Supplementary material for: The Workwell trial: protocol for the process evaluation of a randomised controlled trial of job retention vocational rehabilitation for employed people with inflammatory arthritis
Source: Trials. 2022 Nov 9;23:937. doi: 10.1186/s13063-022-06871-z (PMC9645762; doi:10.1186/s13063-022-06871-z)
Supplement: Supplementary file 3 — Additional file 3. Trial participants’ consent form. [file 13063_2022_6871_MOESM3_ESM.docx]

**Additional File 3: Workwell Intervention: Process evaluation protocol. Hammond et al, 2022.**

|  |  | [Hospital/site heading] |
| --- | --- | --- |

**Patient Screening Number:**

| **S** |  |  |  |  |  |  |
| --- | --- | --- | --- | --- | --- | --- |

**WORKWELL** **CONSENT FORM**

**Title of project: WORKWELL: Testing work advice for people with arthritis**

**Name of researcher: Prof Alison Hammond Please INITIAL all boxes (i.e. do NOT tick)**

1. I confirm that I have read and understand the information sheet dated 9.9.19 **(Version 3**) for the above study. I have had the opportunity to consider the information, ask questions and have had these answered satisfactorily.
2. I understand that my participation is voluntary and that I am free to withdraw at any time, without giving any reason, without my medical care or legal rights being affected.
3. If I do later choose to withdraw from the study, I agree that any data collected up to that point can be kept and used in the study, unless I inform the researchers otherwise.
4. I agree to participate and understand that I will receive a work self-help information pack and I may also be allocated to attend the WORKWELL programme at my Rheumatology/ Therapy department.
5. **Optional:** If I see a therapist as part of the research: I agree to allow one appointment to be audiorecorded by the therapist. I understand that: this will be securely sent to the research team; the therapist will delete their copy; the recording is deleted once transcribed; and anonymised quotes may be given verbatim in reports.
6. **Optional:** if I see a therapist as part of the research: I agree to take part in the face-to-face interview about the work advice I receive. I understand that the interview will be audio-recorded, recordings will be deleted once transcribed and anonymised quotes may be given verbatim in reports.
7. I understand that relevant sections of my medical /therapy notes may be looked at by members of the research team, regulatory authorities or from the NHS Trust, where it is relevant for my taking part in this research. I give permission for these individuals to access my records.
8. I understand that my personal details will be kept confidential and will not be revealed to people outside the research team
9. I agree to my Rheumatology Consultant being informed of my participation in this study.
10. I understand a copy of this form and my contact details will be forwarded by the Research Facilitator/ therapy team at my hospital to the research team at the University of Salford and to the Lancashire Clinical Trials Unit.
11. I understand that my fully anonymised data will be used in research presentations, reports and articles.
12. I agree to take part in the above study.
13. I agree to being contacted in future to **ask about** taking part in a longer-term follow-up for this study and other associated studies. I understand that I can change my mind about this at a later date.

Name of patient:_____________________Date:____________**Signature:**_________________

Name of person

taking consent:______________________ Date:____________ **Signature**:________________

When completed copy x3: 1 for patient; 1 for medical notes; 1 for WORKWELL Trial Manager (UoS); and file original in WORKWELL site file.
