# Supplementary material for: The Workwell trial: protocol for the process evaluation of a randomised controlled trial of job retention vocational rehabilitation for employed people with inflammatory arthritis
Source: Trials. 2022 Nov 9;23:937. doi: 10.1186/s13063-022-06871-z (PMC9645762; doi:10.1186/s13063-022-06871-z)
Supplement: Supplementary file 4 — Additional file 4. Workwell trial: NPT constructs mapped to participants and employer topic guide. [file 13063_2022_6871_MOESM4_ESM.docx]

| **Additional File 4: Workwell trial: NPT constructs mapped to participants and employer topic guide Hammond et al, 2022.** | | |
| --- | --- | --- |
| **Normalisation Process Theory Constructs** | **Participants** | **Participants’ employers** |
| 1. **Coherence** (how the work that defines and organizes work rehabilitation assessment and intervention is understood, made meaningful and invested in at individual and collective levels)    1. Differentiation    2. Communal specification    3. Individual specification    4. Internalisation | Can you describe the work problems you discussed at your first meeting?  Can you describe what happened in the following meetings?  Can you describe the advice or support they gave you for each of your work problems? | What are your views about the work assessment and support provided by the Therapist to your employee?  What types of assistance do you know the therapist provided? |
| 1. **Cognitive participation** (commitment to and engagement of the participant with the intervention)    1. Initiation    2. Enrolment    3. Legitimation    4. Activation | Have you made any practical changes in your workplace since your appointments with the occupational therapist?  If you have made changes, please can you describe the changes you have made at work?  How long did it take you to make the changes?  How helpful or not helpful were these changes?  Have you made any changes in the way you do your work? (Such as taking short breaks, relaxation methods, stress management techniques, disclosing the condition to colleagues)  Can you describe what the relationship was like between you and your occupational therapist?  What support have you received from your employer/line manager to help you stay in work?  Can you describe any support you have received from your work colleagues?  Are there any changes you have made to your home routines to help you stay in work? Did this come about because of having the workwell appointments?  Are there any changes you have made to your social / leisure activities to help you stay in work? Did this come about because of having the workwell appointments?  Are there any other factors which have helped? | How helpful or not were these different elements? (The employer will be asked to expand on their views related to these different elements).  Was there any advice/ assistance that you consider was unnecessary? If yes, what, and why? |
| 1. **Collective action** (the work needed to implement the intervention, and anticipated pay-off (or cost) this work may bring)    1. Interactional workability    2. Relational integration    3. Skillset workability    4. Contextual integration | Can you describe what helped you make these changes?  What were the immediate benefits of making the changes, if any?  What do you think will be the longer-term benefits or losses, if any?  If you have not made changes, please can we explore this further – why do you feel you have not been able to make any practical changes  Can you tell me what the experience of getting to your WORKWELL appointments was like?  What are your views about the way the appointments were delivered? i.e., can you describe how your experiences with the face-to-face / telephone / video appointments? | Was there any advice/ assistance you would have liked to your employee to receive but they did not? If yes, what, and why?  Was there any other advice/ assistance you would have liked to receive as the employee’s line manager/employer? (e.g., practical; resource information; general information about arthritis) |
| 1. **Reflexive monitoring** (Participants’ / employers’ individual and collective on-going formal and informal appraisal of the intervention)    1. Systemisation    2. Communal appraisal    3. Individual appraisal    4. Reconfiguration | Do you think it was appropriate for the Rheumatology team to provide you with help about work issues?  Was the work programme you received right for you? Why? Why not?  Was the investment in terms of your time worthwhile? Why? Why not?  Can you describe any advice / assistance you felt were unnecessary, or would have liked to receive but did not?  What could be improved or done differently?  Are there any other comments you would like to make about the work support provided? | Was there any other advice/ assistance you would have considered beneficial for the employee’s work colleagues/others in the workplace to have received?  Are there any other comments you would like to make about the work support provided? |
