# Supplementary material for: The Workwell trial: protocol for the process evaluation of a randomised controlled trial of job retention vocational rehabilitation for employed people with inflammatory arthritis
Source: Trials. 2022 Nov 9;23:937. doi: 10.1186/s13063-022-06871-z (PMC9645762; doi:10.1186/s13063-022-06871-z)
Supplement: Supplementary file 5 — Additional file 5. Workwell trial: NPT constructs mapped to therapist topic guide. [file 13063_2022_6871_MOESM5_ESM.docx]

| **Normalisation Process Theory Constructs** | **NHS Therapists Interview and Focus Group Topics** | | |
| --- | --- | --- | --- |
|  | **Pre-trial work services at sites (Interview)**  “What work-related advice and support is typically provided for employed people with RA, IA or PsA in your Rheumatology and Therapy service?” | **Training and Mentoring**  **(Interview and Focus Group)**  “What are your views about the VR training programme you attended?” | **Following the Intervention Delivery (Interview and Focus Group)**  “What were your experiences of delivering the trial intervention?” |
| 1. **Coherence** (how the work that defines and organizes work rehabilitation assessment and intervention is understood, made meaningful and invested in at individual and collective levels) | How does therapists describe the pre-trial work-related therapy/ advice provision on site?  What is the referral pathway?  How is the trial intervention similar to/different from usual care?  Who would (most) benefit from the intervention?  What work-related solutions / advice do they commonly provide? | What were the most/ least helpful elements for them?   - Anything should be added or omitted to support training? - What are their views on the VR mentoring received?   What are their views about the training provided in completing the WES-RC?   - Anything else should be added/ removed from the manual - Ease of access/ usability of the WES-RC manual | How/ did they modify the way in which they have delivered the Workwell intervention in practice due to the Covid pandemic and resulting changes in the service provision?  How did they adjust to this as a department/ service?  What remote technologies were available to deliver the intervention and why? |
| 1. **Cognitive participation** (commitment to and engagement of the therapists with the intervention) | Do therapists see value/potential in the intervention?  Do they think there could be barriers and facilitators to expanding work provision? | Have they found the training and experience a worthwhile investment of time?  Do they feel they have the competence/resources to deliver the intervention effectively? | How did therapists accommodate the intervention sessions/follow up actions in their practice? |
| 1. **Collective action** (the work needed to implement the intervention, and anticipated pay-off (or cost) this work may bring) | How compatible is the intervention with the existing service provision?  What work-related solutions / advice do they commonly provide?  What work-related resources do they commonly provide?  Do they refer people to any other work services? If yes, how often?  How long typically do they provide work-related information to a patient for?  Who provide these? | Were they able to make time/ space to attend training/ mentoring?   - Views about the length of the training - Usability of/ access to the WES-RC training manual in daily practice - Utilisation of mentoring - Integration of the training in practice   How confident they felt after treating at least one patient? | How well or not do they think patients engaged with the WORKWELL programme? (Also explored via Focus Groups)  To their knowledge, did any patients allocated to WORKWELL receive any further work-related therapy after completing WORKWELL with them (or any other WORKWELL therapist at their site)? If yes, what happened?  To their knowledge, did any patients allocated to the control group receive any work-related therapy from therapists at their site? If yes, what happened?  What has been the response of the Therapy department to them for providing this work service? (Also explored via Focus Groups)  Has their department’s work advice provision for people with musculoskeletal conditions and arthritis changed during the last 18 months?  How are employed patients work related problems identified in the Rheumatology clinic now?  Do they routinely receive referrals from clinic for patients with work related problems? If yes, how many per month (estimated)?  Do any other members of the Rheumatology team provide work related advice or support? If yes, please describe what this is.  How do they identify what are employed patients’ work- related problems?  Do they routinely use a structured assessment to guide identifying work related problems? |
| 1. **Reflexive monitoring** (Therapists’ individual and collective on-going formal and informal appraisal of the intervention)    1. Systemisation    2. Communal appraisal    3. Individual appraisal    4. Reconfiguration | Perceived effects on patient care and service provision  If this trial is successful, do they think their department/ Rheumatology service would want to expand their work support provision? If yes, how?  Views on time/resources invested in delivery vs impact  What is needed to make it possible to roll out the intervention effectively? (changes to intervention; changes in services/resources needed for delivery) | Perceived impact of the VR training received  Views on time/resources invested in participation in training vs impact  What was good about the training and what could be improved?  **This section is explored following an online presentation of the future plans for the Workwell Digital Learning Platform and Workwell Patient Solutions Toolkit**  **(Focus Group only)**  We are in the process of designing a Workwell digital learning platform to digitise our work rehabilitation training programme and make it freely accessible to therapists who are willing to undertake this training online, in their own time. What do you think about this? Would you use it? How often would you use it? Do you have any suggestions on the content and modes of delivery? (Only explored via Focus Group)  We are also planning to create an online Workwell Solutions Toolkit for people with arthritis who are struggling at work, based on the common problems identified by patients and therapists within the Workwell Trial. What do you think about this? Do you think this would be a useful tool to share with your patients? Would you like to be part of an expert panel to inform this? (Only explored via Focus Group) | Reflecting on pre and post Covid pandemic, how did this impact on their delivery of the trial intervention?  How did they find delivering the intervention remotely? Is this something they would like to continue post-covid?  Reflecting on their current service and the WORKWELL programme:  Would they consider implementation of the WORKWELL programme in their Therapy and Rheumatology service for people with inflammatory arthritis and other conditions? (Also explored via Focus Groups)  What factors might help his? What barriers might hinder this?  Are there any other comments they would like to make about: the WORKWELL intervention? their delivery of the intervention? implementation in future? (Also explored via Focus Groups) |
